# Cytosolic nucleic acid sensing and mitochondrial transcriptomic changes as early triggers of metabolic disease in db/db mice

**DOI:** 10.1007/s00335-023-10026-z

**Published:** 2023-11-18

**Authors:** Agnieszka H. Ludwig-Słomczyńska, Michał T. Seweryn, Jerzy Wiater, Agnieszka Borys, Anna Ledwoń, Magdalena Druszczyńska, Magdalena Łabieniec-Watała, Grzegorz J. Lis, Paweł P. Wołkow

**Affiliations:** 1 Center for Medical Genomics OMICRON, Jagiellonian University Medical College, Kraków, Poland; 2 Biobank Lab, Faculty of Biology and Environmental Protection, University of Lodz, Lodz, Poland; 3 Department of Histology, Faculty of Medicine, Jagiellonian University Medical College, Kraków, Poland; 4 Department of Immunology and Infectious Biology, Faculty of Biology and Environmental Protection, University of Lodz, Lodz, Poland; 5 Department of Medical Biophysics, Faculty of Biology and Environmental Protection, University of Lodz, Lodz, Poland

## Abstract

Animal models of diabetes, such as db/db mice, are a useful tool for deciphering the genetic background of molecular changes at the initial stages of disease development. Our goal was to find early transcriptomic changes in three tissues involved in metabolism regulation in db/db mice: adipose tissue, muscle tissue and liver tissue. Nine animals (three per time point) were studied. Tissues were collected at 8, 12 and 16 weeks of age. Transcriptome-wide analysis was performed using mRNA-seq. Libraries were sequenced on NextSeq (Illumina). Differential expression (DE) analysis was performed with edgeR. The analysis of the gene expression profile shared by all three tissues revealed eight upregulated genes (*Irf7, Sp100, Neb, Stat2, Oas2, Rtp4, H2-T24* and *Oasl2*) as early as between 8 and 12 weeks of age. The most pronounced differences were found in liver tissue: nine DE genes between 8 and 12 weeks of age (*Irf7, Ly6a, Ly6g6d, H2-Dma, Pld4, Ly86, Fcer1g, Ly6e* and *Idi1*) and five between 12 and 16 weeks of age *(Irf7, Plac8, Ifi44, Xaf1* and *Ly6a*) (adj. *p*-value < 0.05). The mitochondrial transcriptomic profile also changed with time: we found two downregulated genes in mice between 8 and 12 weeks old (*Ckmt2* and *Cox6a2*) and five DE genes between 12 and 16 weeks of age (*Mavs, Tomm40L, Mtfp1, Ckmt2* and *Cox6a2*). The KEGG pathway analysis showed significant enrichment in pathways related to the autoimmune response and cytosolic DNA sensing. Our results suggest an important involvement of the immunological response, mainly cytosolic nucleic acid sensing, and mitochondrial signalling in the early stages of diabetes and obesity.

**Supplementary Information:**

The online version contains supplementary material available at 10.1007/s00335-023-10026-z.

## Introduction

Animal models play an important role in understanding the aetiology of diseases as well as their multisystemic effects (Okechukwu 2018). In this study, we used a genetically induced mouse model of type 2 diabetes with db/db mice, which have a mutation in the leptin receptor gene (LepR) and are characterised by obesity, decreased insulin receptor sensitivity and elevated levels of blood glucose (Burke et al. 2017).

It is already known that metaflammation, a process in which macrophages become polarised towards pro-inflammatory phenotypes, thus resulting in the production of pro-inflammatory cytokines, plays a crucial role in the development of obesity. Moreover, according to recent studies, the activation of the immune response stems from the accumulation of nucleic acids and their recognition by damage-associated molecular patterns (DAMPs). It has also been shown that cGas and STING activation is increased in mice fed a high-fat diet and that it is due to the release of mitochondrial DNA in adipose tissue.

Our goal was to find very early transcriptional changes during the development of obesity in the db/db mouse model. We analysed mice at 8, 12 and 16 weeks of age to track changes in the transcriptomic profile in three metabolically active tissues: adipose tissue, liver tissue and muscle tissue. We confirmed that sterile inflammation is the underlying cause of diabetes and obesity in db/db mice and showed that the activation of the cGas-STING pathway and the resulting upregulation of *Irf7* takes place as early as at 8 weeks of age. Moreover, we found that as obesity develops after 12 weeks of age, the activation of *Irf7* is muted, possibly to regulate the metabolic response, as loss of *Irf7* was shown to decrease glucose tolerance. This observation also confirms that the activation of the immune response is a very early event in the development of the disease development. Tissue-wise, the most pronounced changes were observed in liver tissue, which shows that it is not only immune cells and adipocytes that contribute to obesity-mediated pathogenesis. Our data show the whole transcriptomic profile of young obese mice and underline the importance of sterile inflammation as the earliest cause of the disease.

## Materials and methods

### Animals and tissue collection

Nine Dock7 <m>  + / + Lepr <db> /J males (db/db mice) were purchased from Charles River. The animals were acclimated for 2 weeks at the age of 6 weeks. The mice were kept in temperature-controlled rooms 21 (± 0.5 to ± 5%), humidity 55% on a 12-h light/12-h dark cycle and had ad libitum access to pelleted food and filtered water. The animals were randomly allocated into groups and examined at three stages of life: 8 weeks, 12 weeks and 16 weeks. Three animals were analysed at each time point. On the day of sacrifice, liver, muscle and adipose tissue were immediately snap-frozen or collected in RNAlater solution and stored at − 80 °C. All procedures were approved by the Local Ethics Commission for Animal Experiments in Łódź, Poland.

### RNA isolation and sequencing

Total RNA was isolated using a Maxwell 16 Instrument (Promega) from the following groups: 8-week-old db/db mice (*n* = 3; animals A, B, C), 12-week-old mice (*n* = 3; animals D, E, F) and 16-week-old mice (*n* = 3; animals G, H, I). The quality and quantity were checked with Quantus (Promega) and Tape Station (Agilent). 500 ng of total RNA was used to prepare libraries according to SENSE mRNA-Seq Library Preparation Kit (Lexogen). The libraries were sequenced on NextSeq (Illumina).

### Bioinformatic and statistical analysis

The bioinformatic analysis consisted of trimming using the Cutadapt tool (Martin 2011), mapping sequences to the GRCm38 mouse reference genome with the STAR aligner (Dobin et al. 2013) and counting the mapped mRNA reads using HT-Seq (Putri et al. 2022). Differential expression analysis was performed with the aid of edgeR package in R (Robinson et al. 2010). Analysis of the KEGG (Kyoto Encyclopedia of Genes and Genomes) and GO (Gene Ontology) pathways was also performed. Analysis of mitochondrial gene expression was performed on genes reported to be associated with mitochondrial functioning listed in MitoCarta (Rath et al. 2021).

### Immunofluorescence and imaging

Frozen sections were air-dried for several minutes and fixed in 4% PFA (for 15 min), permeabilised in 1% (v/v) Triton-X in PBS (for 10 min) and blocked with 1% BSA blocker (for 1.5 h). An overnight incubation with primary antibody (cGAS—#703149, Invitrogen, 1:200, + 4 °C or IRF7—#MA541165, Thermo Fischer, 1:100, + 4 °C) was followed by secondary antibody incubation (#96886, Abcam, conjugated with DyLight 650, 1:500, 2 h, RT). The sections were co-stained with MitoView (#70054, Biotium, 1:1000) or dsDNA Dye (#E2670, Promega, 1:400) for 30 or 20 min, respectively. Immunofluorescence-labelled sections were mounted with VectaShield® HardSet™ Mounting Medium with DAPI (#H-1500-10, Vector Laboratories) and analysed on Olympus FluoView1200 scanning confocal laser microscope using the 40 × or 60x (oil immersion) objective lens. A 473-nm diode laser, a 635-nm diode laser and a 405-nm diode laser were used to excite green (MitoView, dsDNA Dye), far-red (DyLight 650) and blue (DAPI) fluorescence, respectively. At least three images per sample were collected. Each image was divided into 36 ROIs. These were used to calculate the fluorescence intensity per image.

For lipid visualisation, the sections were fixed in 4% PFA, washed with dH2O, rinsed with 60% isopropanol and stained with 4.5 g/l Oil Red O (#O0625-25G, Sigma Aldrich) for 15 min. Excess dye was removed by 60% isopropanol rinsing, followed by a running tap water wash. The nuclei were counterstained with Harris haematoxylin and slides were mounted. High-resolution bright-field images were taken (under 20 × magnification) using an Olympus IX83 microscope and were analysed using CellSens Dimension software (Olympus). Three images per sample were collected. Each image was divided into 36 ROIs. At least 27 ROIs were used to calculate the amount of lipid accumulation by analysing the stained surface area in μm^2^.

## Results

The aim of the study was to observe the early events of diabetes and obesity development at the transcriptomic level. Three metabolically important tissues were analysed: adipose tissue, liver tissue and muscle tissue.

**Fig. 1:**
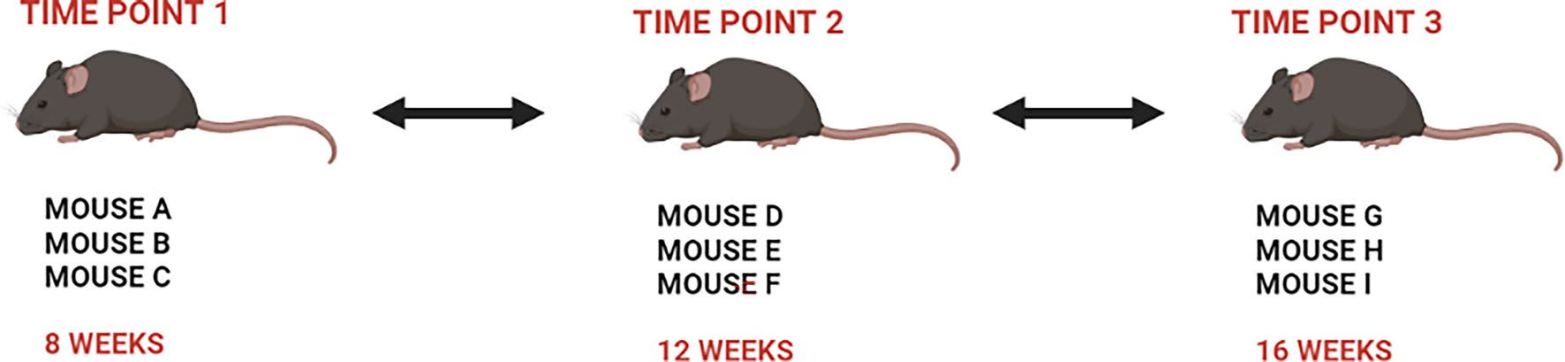
The design of the experiment. The study focused on 3 time points each representing different animal age. Time point 1: 8-week-old animals, time point 2: 12-week-old animals, time point 3: 16-week-old animals. Three metabolically active tissues: adipose tissue, liver tissue and muscle tissue were analyzed

Nine db/db mice were analysed over an 8-week period. Time point 1 represents 8-week-old animals (marked as A, B and C). Time point 2 represents the transcriptional profile of 12-week-old animals (mice labelled D, E, and F), while time point 3 represents 16 week-old mice (animals G, H, I). A diagram of the experiment is shown in Fig. 1.

The animals were weighed weekly and their blood glucose levels (fasting state) and other biochemical parameters were checked, as shown in Table S1.

RNA profiles of adipose tissue, muscle tissue and liver tissue were analysed at three time points. The RNA expression profiles were specific to each tissue and maintained throughout the analysed time points, as shown by the clusters in Fig. S1.

### Analysis of the gene expression profile shared by all three tissues over the course of the experiment

Regardless of the transcriptional uniqueness of the tissues, our goal was to check whether there is a trend in gene expression which would be shared by all three tissues; thus, we searched for genes with different expression between different time points in all the selected tissues. We identified eight statistically significant genes which were upregulated between time points 2 and 1 (Table 1). No differentially expressed genes were found between time points 3 and 2.

**Table 1:** The gene expression profile shared by all three tissues analysed between time points 2 and 1

Gene	logFC	Average expression	*p*-value	Adjusted *p*-value
*Irf7*	2.49	3.75	2.56E−08	0.0003
*Sp100*	1.14	4.08	9.09E−06	0.0330
*Neb*	1.83	7.15	1.60E−05	0.0397
*Stat2*	1.62	4.01	1.07E−05	0.0330
*Oas2*	2.15	1.69	7.79E−06	0.0330
*Rtp4*	2.32	3.68	2.26E−05	0.0466
*H2-T24*	1.70	2.28	2.68E−05	0.0474
*Oasl2*	2.18	2.79	3.39E−05	0.0525

**Fig. 2:**
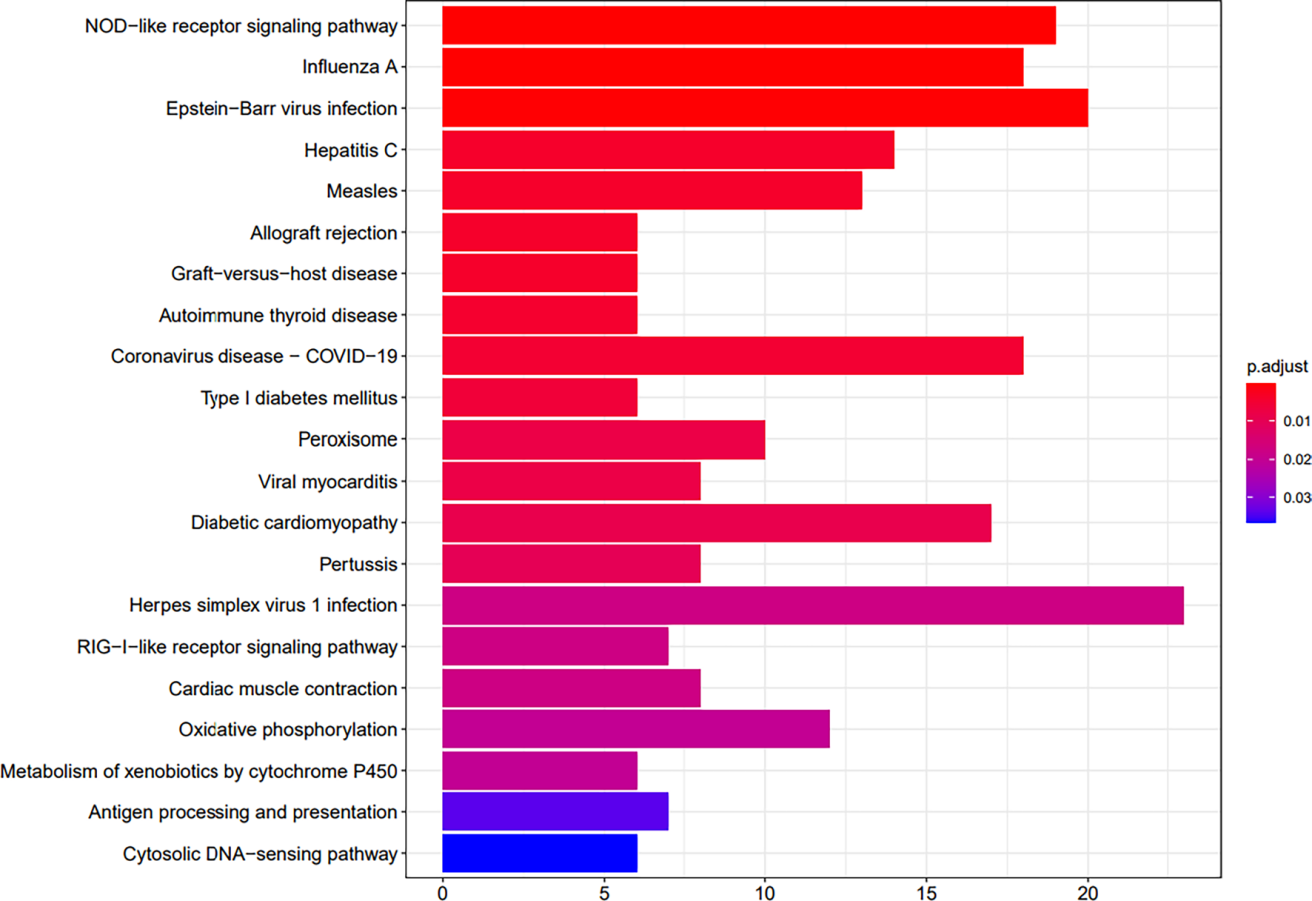
KEGG pathways significantly enriched between time points 2 and 1 (12- vs 8-week-old mice) in all tissues analysed

Both analyses, however, yielded a substantial number of nominally significant genes with differential expression. These were subjected to KEGG pathway analysis, which led to the discovery of 21 pathways between time points 2 and 1 (Fig. 2) and two between time points 3 and 2: oxidative phosphorylation (mmu00190) and diabetic cardiomyopathy (mmu05415) (both were also enriched in the first analysis). The GO enrichment for these comparisons is shown in Table S2.

### Analysis of unidirectional, tissue-specific changes in gene expression over the course of the experiment

Next, we wanted to check which tissue undergoes the greatest unidirectional change in gene expression over the course of the experiment. The PCA analysis showed a trend for directionality for liver tissue only (Fig. S2), where we found 507 genes with unidirectional change (Table S3). Interestingly, the KEGG pathway analysis revealed no common pathway for them. However, 30 genes were associated with mitochondria (Table 2).

**Table 2:** Mitochondrial genes with unidirectional change in liver tissue

Gene	logFC	Average expression	*p*-value	Adjusted *p*-value
*Mavs*	0.60	5.72	8.69E−06	0.0133
*Cry1*	0.97	2.54	5.45E−05	0.0194
*Tomm40L*	0.77	2.71	0.0001	0.0235
*Ckmt2*	− 2.08	2.36	0.0001	0.0235
*Lap3*	− 0.45	5.10	0.0001	0.0235
*Crat*	0.52	7.97	0.0001	0.0235
*Rdh13*	0.58	1.88	0.0001	0.0235
*Tstd3*	− 0.58	3.02	0.0002	0.0258
*Cox6a2*	− 1.84	1.77	0.0002	0.0280
*Ccdc90b*	− 0.53	1.96	0.0002	0.0284
*Mtrf1*	− 0.53	2.82	0.0002	0.0294
*Mrpl1*	− 0.47	3.38	0.0003	0.0294
*Fundc2*	− 0.50	3.66	0.0003	0.0295
*Yars2*	− 0.53	2.65	0.0005	0.0314
*Slirp*	− 0.56	4.04	0.0005	0.0313
*Cox8b*	− 1.73	2.71	0.0006	0.0328
*Nadk2*	− 0.51	4.12	0.0006	0.0328
*Crot*	− 0.43	6.13	0.0006	0.0328
*Ssbp1*	− 0.46	2.83	0.0007	0.0340
*Pnpt1*	− 0.46	3.47	0.0007	0.0340
*Rdh14*	− 0.53	2.88	0.0010	0.0384
*Atpaf1*	− 0.35	3.80	0.0011	0.0403
*Yme1l1*	− 0.44	5.08	0.0012	0.0405
*Nars2*	− 0.49	2.59	0.0013	0.0421
*Cox10*	0.38	4.00	0.0013	0.0421
*Alas1*	0.66	7.28	0.0013	0.0399
*Pmpcb*	− 0.35	5.74	0.0013	0.0418
*Mff*	− 0.32	5.78	0.0013	0.0419
*Mtif2*	− 0.39	3.66	0.0015	0.0441
*Tomm70a*	− 0.42	5.79	0.0015	0.0436

The analysis of adipose tissue and muscle did not show any statistically significant genes with unidirectional change in expression, while the pathway analysis found two enriched pathways for adipose tissue (oxidative phosphorylation [mmu00190] and diabetic cardiomyopathy [mmu05415]) and seven pathways for muscle (protein digestion and absorption [mmu04974], platinum drug resistance [mmu01524], ECM-receptor interaction [mmu04512], focal adhesion [mmu04510], amoebiasis [mmu05146], African trypanosomiasis [mmu05143], and human papillomavirus infection [mmu05165]) (Table S4).

### Analysis of tissue-specific changes between the time points

Finally, we decided to compare the expression profiles between the time points within each tissue. No statistically significant differences in gene expression were found in the adipose tissue. We found five genes in the muscle tissue that differed between time points 3 and 2, while no changes were found between time points 2 and 1 (Table 3). In the liver tissue, we identified nine genes that differed between time points 2 and 1, six that differed between time points 3 and 1, and five that differed between time points 3 and 2 (Table 3).

**Table 3:** Statistically significant genes in the per tissue comparisons

Gene	logFC	Average expression	*p*-value	Adjusted *p*-value
**LIVER 2 vs 1**				
*Irf7*	3.28	4.77	1.27E−07	0.001
*Ly6a*	2.31	3.93	3.72E−06	0.022
*Ly6g6d*	2.53	1.24	1.67E−05	0.044
*H2-Dma*	1.73	2.96	1.97E−05	0.044
*Pld4*	2.42	1.33	2.09E−05	0.044
*Ly86*	1.39	4.47	2.52E−05	0.044
*Fcer1g*	1.63	1.87	2.61E−05	0.044
*Ly6e*	2.18	8.17	3.03E−05	0.045
*Idi1*	− 2.02	2.99	3.58E−05	0.047
**LIVER 3 vs 2**				
*Irf7*	− 3.33	4.77	1.00E−07	0.001
*Plac8*	− 2.06	2.39	4.44E−06	0.026
*Ifi44*	− 3.77	1.05	1.14E−05	0.033
*Xaf1*	− 2.54	3.67	1.38E−05	0.033
*Ly6a*	− 2.02	3.93	1.31E−05	0.033
**LIVER 3 vs 1**				
*C9*	− 1.84	7.68	2.36E−06	0.028
*Neb*	− 2.06	4.22	5.97E−06	0.035
*Cdkn1a*	3.50	3.70	2.56E−05	0.050
*Mup-ps13*	− 2.95	1.20	2.53E−05	0.050
*Rbm14*	1.87	3.16	2.26E−05	0.050
*Tsku*	1.78	6.53	1.52E−05	0.050
**MUSCLE 3 vs 2**				
*Irf7*	− 3.53	1.44	2.50E−07	0.003
*Rtp4*	− 2.42	1.92	3.57E−06	0.015
*Xaf1*	− 1.98	2.97	2.77E−06	0.015
*Oasl2*	− 2.85	1.51	7.62E−06	0.019
*Pfkfb3*	− 2.27	6.83	7.63E−06	0.019

When we looked specifically into mitochondrial gene expression, we found two genes whose expression was significantly downregulated between time points 2 and 1, and five differentially regulated genes between time points 3 and 2 (Table 4).

**Table 4:** Statistically significant mitochondrially associated genes in liver tissue

Gene	logFC	Average expression	*p*-value	Adjusted *p*-value
**LIVER 2 vs 1**				
*Ckmt2*	− 5.36	2.36	4.43E−05	0.0221
*Cox6a2*	− 5.42	1.77	2.77E−05	0.0221
**LIVER 3 vs 2**				
*Mavs*	1.33	5.73	0.0001	0.0318
*Tomm40L*	1.80	2.71	0.0001	0.0318
*Mtfp1*	1.85	2.92	0.0001	0.0318
*Ckmt2*	− 4.94	2.35	0.0001	0.0318
*Cox6a2*	− 5.69	1.77	1.33E−05	0.0132

The KEGG pathway analysis for adipose tissue showed a massive immunologic response to viral infection. Pathways that were common to both analyses (time points 2 vs 1, and time points 3 vs 2) included Epstein-Barr virus, coronavirus, hepatitis C, measles, herpes simplex virus 1, type 1 diabetes, RIG-like receptor signalling and human papillomavirus infection (Table S5). There were also several pathways specific to particular time points. For time points 2 vs 1, we found the cytosolic DNA sensing pathway, oxidative phosphorylation, cardiac muscle contraction, diabetic cardiomyopathy, DNA adducts and metabolism of xenobiotics by cytochrome P450. Only three pathways were uniquely characteristic of time points 3 vs 2: the pathways for toxoplasmosis, cell adhesion molecules and C-type lectin receptor signalling (Table S5).

In muscle tissue, pathways involved in the autoimmune response were the most enriched both in the analysis of time points 2 vs 1 and time points 3 vs 2. Pathways specific to time points 2 vs 1 included the anti-viral response (Kapsi sarcoma-associated herpesvirus infection, herpes simplex virus 1 infection, human immunodeficiency virus 1 infection, and human papillomavirus infection), the NOD-like receptor signalling pathway, cell adhesion and cell senescence. Interestingly, more specific changes were observed when we compared time points 3 and 1, namely enrichment of protein digestion and adsorption, focal adhesion, the AGE-RAGE signalling pathway in diabetic complications and the TNF signalling pathway, phagosome, apoptosis and cytokine-cytokine receptor interaction (Table S5).

**Fig. 3:**
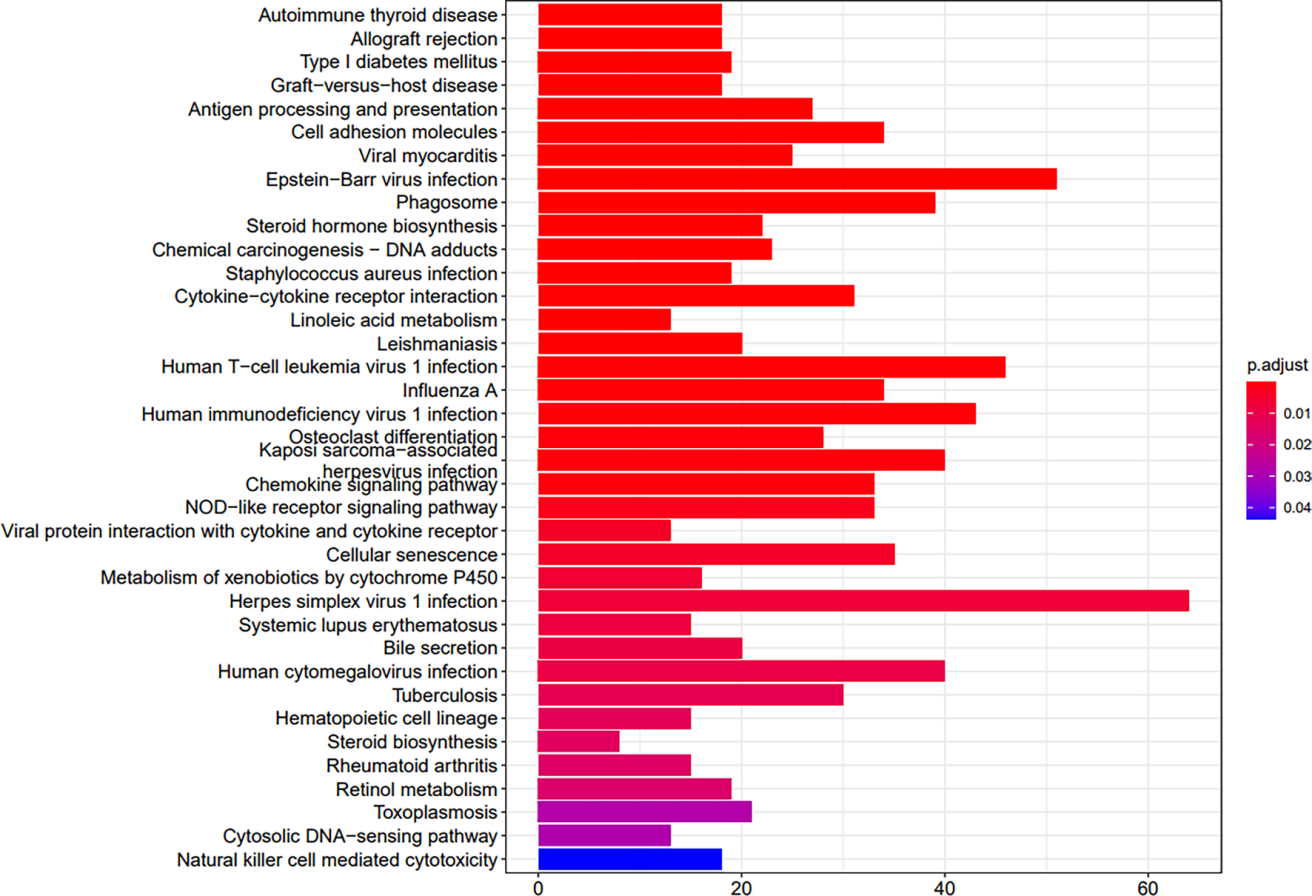
KEGG pathways significantly enriched between time points 2 and 1 (12- vs 8-week-old mice) in liver tissue

In liver tissue, similarly to muscle, the pathways specific to time points 3 vs 2 fully overlapped with those from time points 2 vs 1. These were natural killer cell mediated cytotoxicity, antigen processing and presentation, graft-versus-host disease and autoimmune responses (autoimmune thyroid disease and type 1 diabetes mellitus). The pathways characteristic of time points 2 vs 1 were those associated with immunologic responses (Leishmaniosis, toxoplasmosis and *Staphylococcus aureus*, human cytomegalovirus, human T-cell leukaemia virus 1 and Kaposi sarcoma-associated herpes virus infection), the NOD-like receptor pathway, cytosolic DNA sensing, cytokine-cytokine receptor interaction and the chemokine pathway and pathways involved in steroid biosynthesis and metabolism (Fig. 3 and Table S5).

### Imaging

Since most of the transcriptomic analyses showed that the immunologic response (response to viral infection and cytosolic DNA sensing) and mitochondrially associated genes play a key role in the development of diabetes, we decided to perform mitochondrial imaging and immunostaining for Irf7 and cGas in the liver and muscle tissues of all mice studied.

**Fig. 4:**
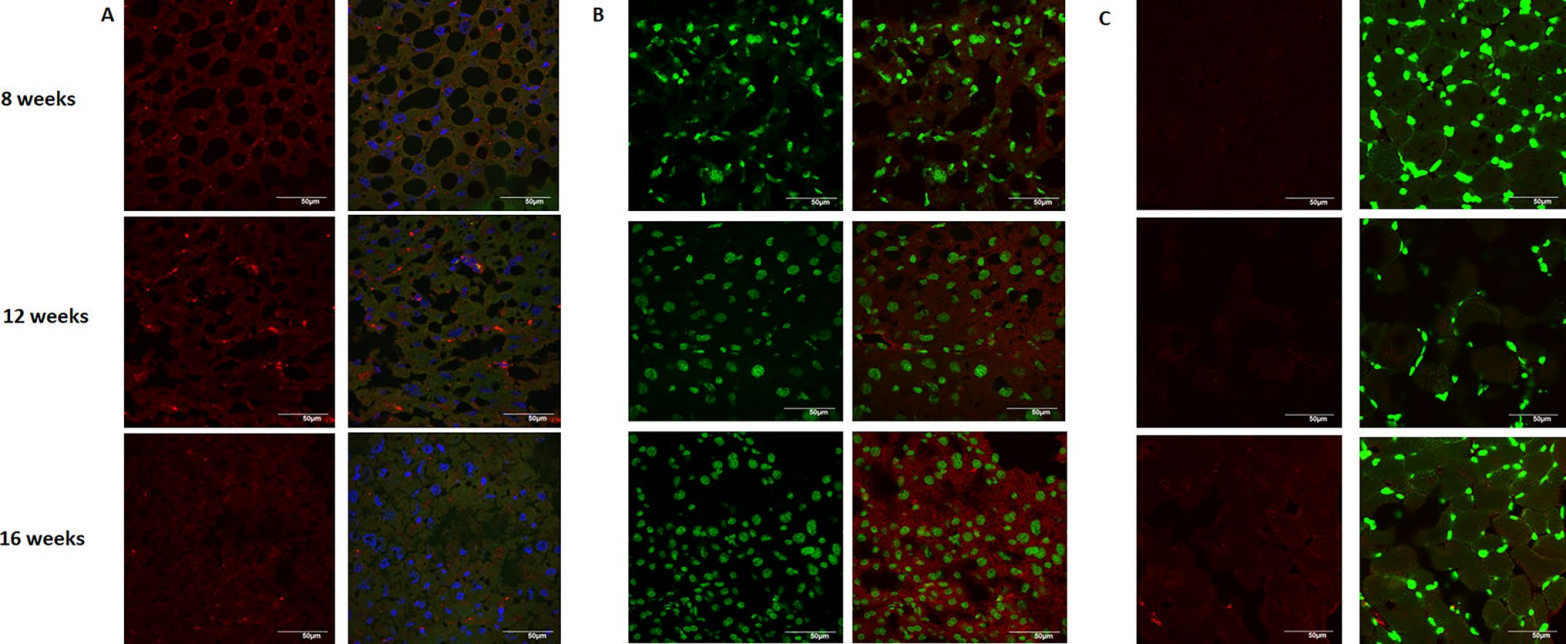
Expression of Irf7 in liver tissue (**A**), cGas in liver tissue (**B**) and cGas in muscle (**C**) (in red) at different time points. Sections were stained with MitoView (for Irf7) or dsDNA Dye (for cGAS) (in green)

The pattern of Irf7 protein expression reflected that observed at the RNA level, as it increased between 8 and 12 weeks of age and decreased between 12 and 16 weeks of age. This trend, even though not statistically significant (time point 1 vs 2 *p* = 0.302, time point 2 vs 3 *p* = 0.155 in liver tissue; time point 1 vs 2 *p* = 0.385, time point 2 vs 3 *p* = 0.698 in muscle tissue) was found in both liver and muscle tissue (Fig. 4A).

The expression pattern of cGas showed a steady statistically insignificant increase in liver tissue (time point 1 vs 2 *p* = 0.801, time point 2 vs 3 *p* = 0.881) (Fig. 4B), while it tended to decrease in muscle between 12 and 16 weeks of age (time point 1 vs 2 *p* = 0.469, time point 2 vs 3 *p* = 0.037) (Fig. 4C).

We also performed Oil Red O staining to check liver lipid accumulation. Our analysis revealed a non-statistically significant decrease in lipid staining at 12 weeks of age compared to 8 weeks of age (*p* = 0.078). Lipid accumulation increased between 12 and 16 weeks of age (*p* = 0.132).

## Discussion

Our study aimed to find early transcriptomic changes in three tissues involved in metabolism regulation in db/db mice: adipose tissue, muscle tissue and liver tissue. Our results clearly show that a dysregulated immune response along with mitochondrial dysfunction are fundamental to the development of obesity and diabetes as early as at 12 weeks of age.

In our analysis of all sequenced tissues, we found that dysregulation of *Irf7,* a master regulator of the interferon response, is the major driver of the development of diabetes. Other upregulated genes between ages 8 and 12 weeks were *Oas2*, *Stat2*, *Oasl2* and *Sp100*, all of which are transcriptional targets of Irf7 that are also activated by foreign DNA or RNA particles. Both *Oas2* and *Stat2* can be activated by dsRNA, leading to the degradation of viral RNA and the activation of RIG-I pattern recognition receptors (Choi et al. 2015; Li et al. 2022). *Oasl*, a member of the *Oas* family, also interacts with RIG-I and enhances antiviral activity. It has also been shown to act as a negative regulator of the cGAS-STING signalling pathway, which is activated by foreign DNA (Ghosh et al. 2019). Another upregulated gene, *Sp100* is a known inhibitor of double-stranded DNA viruses, e.g. HSV (Stepp et al. 2013). This corroborates the observation that between 8 and 12 weeks of age, the response to the development of diabetes and obesity depends on pattern recognition receptors (PRRs) and is interferon-centred.

We noted an upregulation of *Irf7* in liver tissue between 8 and 12 weeks of age, along with increased expression of cluster of genes known to be induced by it – *Ly6a*, *Ly6e*, *Ly6g6d* – all of which are located next to MHC genes (Khodadoust et al. 1999; Long et al. 2011; Wahadat et al. 2018). The upregulation of Irf7 between the two time points was also seen at the protein level. Moreover, we noted an upregulation of *Pld4,* a phospholipase with 5'- > 3' DNA exonuclease activity, which can digest single-stranded DNA (Gavin et al. 2018). *Pld4* expression is also associated with M1 macrophage expression, which increases the inflammation response (Gao et al. 2017). *Pld4*-deficient mice have inflammatory disease and an exaggerated TLR9 response, while *Pld4*- and *Pld3*-knockdown mice die before the age of 21 days due to lethal liver inflammation (Gavin et al. 2021). We believe that *Pld4* upregulation results from an increase of cytosolic nucleic acids, which activate TLR9 PAMP. This cytosolic DNA is sensed by the cGAS-STING pathway, which is also upregulated by IFN. In fact, our immunofluorescence experiment showed a steady increase of cGAS expression in the cytoplasm of liver samples. The only gene which was downregulated between 8 and 12 weeks of age was *Idi1*. *Idi1* is an enzyme necessary for cholesterol synthesis. Its downregulation might result from an increase in ROS production and lead to lower cholesterol synthesis (Sun et al. 2014). Our Oil Red O staining showed a non-statistically significant decrease in lipid staining in liver at the age of 12 weeks compared to 8-week-old mice.

It is interesting that the interferon-associated immune response is muted after 12 weeks of life. We saw a downregulation of *Irf7* expression along with its target genes, *Ly6a* and *Ifi44*, which are known to inhibit viral replication (Hallen et al. 2007; Busse et al. 2020). This is likely a way to manage increased inflammation. It has been shown that *Irf7* KO ob/ob mice gain less weight than wild-type controls (Wang et al. 2013). The downregulation of Irf7 was also seen in immunofluorescence analysis even though the change was not statistically significant. Moreover, we observed a decrease in *Xaf1*, which is known to contribute to the stabilisation of Irf7 (Jeong et al. 2018). *Xaf1* is also an inducer of apoptosis inhibited by another Ifi family member, Ifi6 (Qi et al. 2015). We also found a decrease in the expression of *Plac8*, which is believed to regulate thermogenesis in brown fat tissue and to influence the size of white adipose tissue cells (Jimenez-Preitner et al. 2011; Lee et al. 2018).

A similar effect was found in muscle tissue, where *Irf7* and *Xaf1* are downregulated in 16-week-old mice (compared to 12-week-old mice). The downregulation of *Rtp4*, the interferon stimulated gene (ISG), might stem from lower *Irf7* expression. However, *Rtp4* is also known to act as a negative regulator of TBK1 and IFN (He et al. 2020). The results also show downregulation of *Oasl2,* which may reduce RIG activation, but can also serve as an activator of the cGAS-STING signalling pathway. In fact, our immunofluorescence analysis show an upregulation of cGAS in muscle. Finally, our results indicate a downregulation of *Pfkfb3*, which is a rate-limiting enzyme responsible for commitment towards glycolysis. It is an activator of 6-phosphofructo-1-kinase. Xiang et al. showed that elevated glycolytic metabolism in muscle tissue leads to reduced obesity and IR, thus its downregulation would exacerbate disease development. *Pfkfb3* also leads to increased lipid metabolism (Xiang et al. 2021). Moreover, Zhu et al. showed that haematopoietic cell specific disruption of *Pfkfb3* aggravates the effects of HFD and leads to increased inflammation (Zhu et al. 2021).

The observed results are closely associated with mitochondrial functioning. The analysis of MitoCarta genes revealed an upregulation of *Mavs*, a mitochondrial antiviral factor, between 12 and 16 weeks of age. Mavs is an adaptor protein activated by extracellular RNA and responsible for the downstream activation of the TBK1 and IRF3 pathways. Moreover, we found increased expression of the mitochondrial transporter *Tomm40L*, which interacts with Mavs and recruits IRF3 to the mitochondria (Jacobs and Coyne 2013), inducing IRF3 phosphorylation, dimerisation and nuclear translocation and allowing a full interferon response (Liu et al. 2015). Moreover, under normal conditions, Mavs interacts with mitofusin Mfn-1 and inhibits Mavs-mediated induction of type I IFN (Sandhir et al. 2017). This interaction is apparently distorted, as we found upregulation of *Mtfp1*, which is responsible for mitochondrial fission and increased cell death (Morita et al. 2017; Aung et al. 2017).

The changes in mitochondrial functioning are also evident from the downregulation of *Cox6a2* and *Ckmt2*. *Cox6a2* is part of complex IV of the electron transport chain. Mice lacking *Cox6a2* have elevated energy expenditure (likely due to increased uncoupling via UCPs), reduced complex IV activity and increased ROS production (Quintens et al. 2013; Nagai et al. 2021). *Ckmt2* synthesises creatine phosphate (*PCr*) from creatine and ATP produced by mitochondria. *PCr* is a better transporter of high-energy phosphate. It also liberates mitochondrial ADP to increase the rate of mitochondrial respiration. However, it has also been found that in the futile creatine cycle, a process in which creatine is phosphorylated and then dephosphorylated, energy is dissipated without being used for any mechanical work (Greenhill 2021). This affects thermogenesis, which is an important factor in countering obesity and diabetes. Downregulation of *Ckmt2* might thus disturb thermogenesis as well as decrease mitochondrial membrane potential and increase ROS production (Park et al. 2021). On the other hand, *PCr* depletion has recently been shown to decrease ATP levels and NLRP3 inflammasome activation (Billingham et al. 2022).

In conclusion, the sterile inflammation associated with severe mitochondrial metabolic changes is the underlying reason for the development of diabetes and obesity in db/db mice as early as in 12-week-old animals. This is consistent with the hypothesis that the mitochondrial genome contributes to the aetiology and progression of inflammation [lately reviewed (Marchi et al. 2022)].

### Supplementary Information

Below is the link to the electronic supplementary material.

Supplementary file1 (TIF 14 KB)

Supplementary file2 (PDF 5 KB)

Supplementary file3 (PDF 5 KB)

Supplementary file4 (PDF 5 KB)

Supplementary file5 (PNG 67 KB)

Supplementary file6 (PNG 41 KB)

Supplementary file7 (PDF 507 KB)

Supplementary file8 (PDF 393 KB)

Supplementary file9 (XLS 46 KB)

Supplementary file10 (XLSX 55 KB)

Supplementary file11 (ODS 4 KB)

Supplementary file12 (ODS 20 KB)

## Data Availability

The data sets generated and analysed during the current study are available from the corresponding authors upon request.
