# Supplementary figures and images for: Cytosolic nucleic acid sensing and mitochondrial transcriptomic changes as early triggers of metabolic disease in db/db mice
Source: Mamm Genome. 2023 Nov 18;35(1):68–76. doi: 10.1007/s00335-023-10026-z (PMC10884043; doi:10.1007/s00335-023-10026-z)

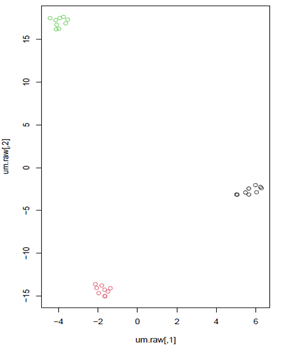

Supplement: Supplementary file 1 — Supplementary file1 (TIF 14 KB) [file 335_2023_10026_MOESM1_ESM.tif]

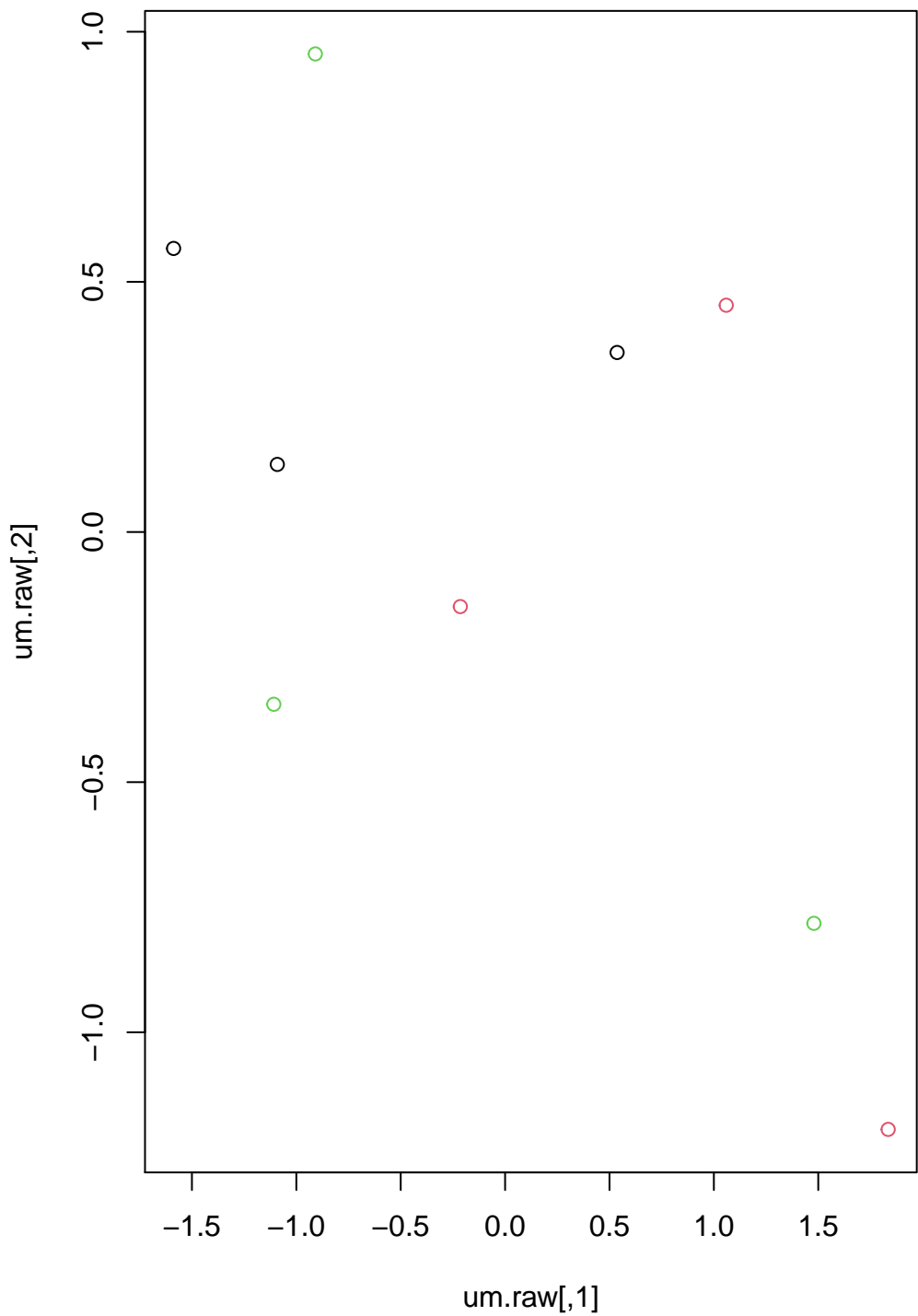

Supplement: Supplementary file 2 — Supplementary file2 (PDF 5 KB) [file 335_2023_10026_MOESM2_ESM.pdf]

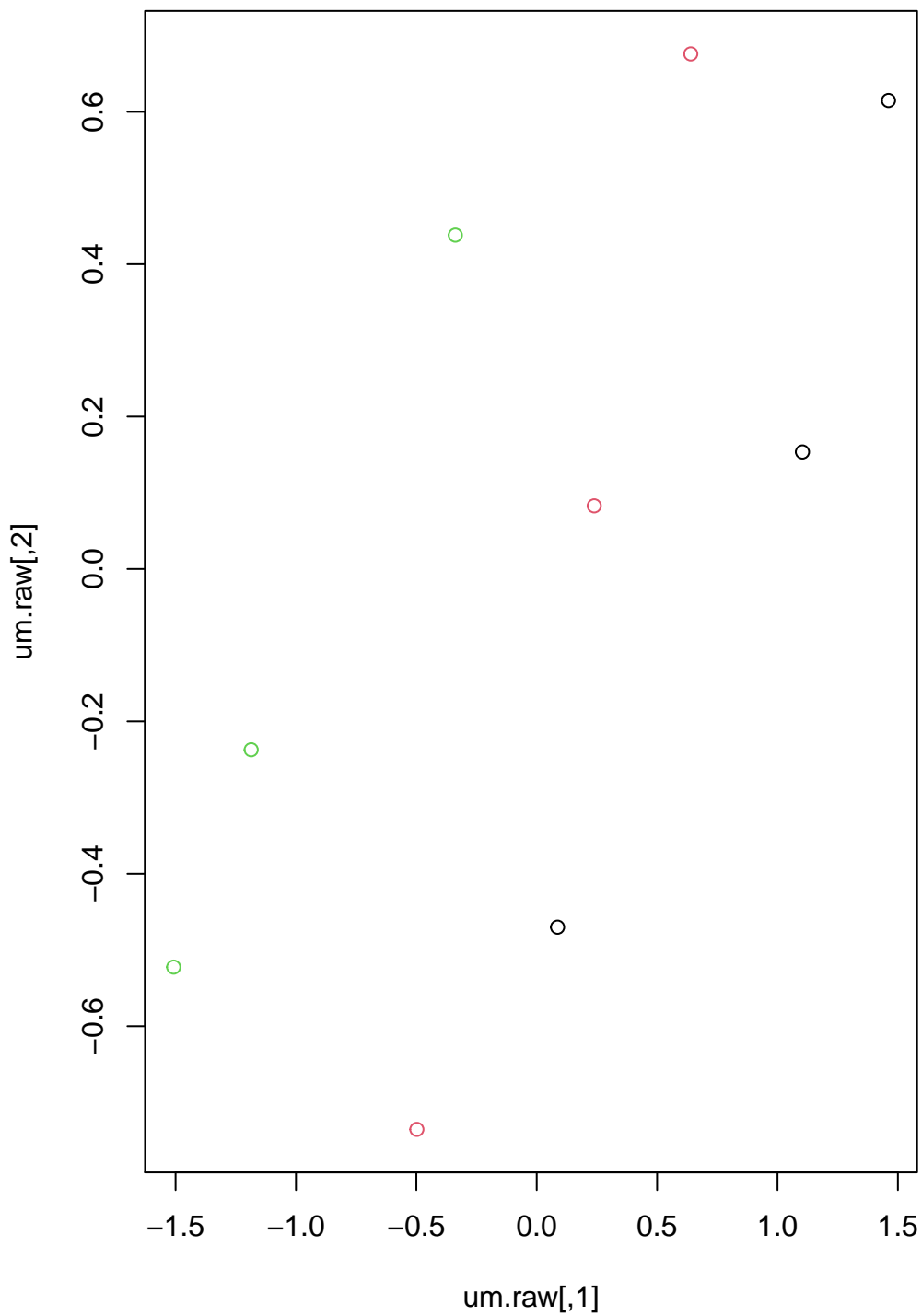

Supplement: Supplementary file 3 — Supplementary file3 (PDF 5 KB) [file 335_2023_10026_MOESM3_ESM.pdf]

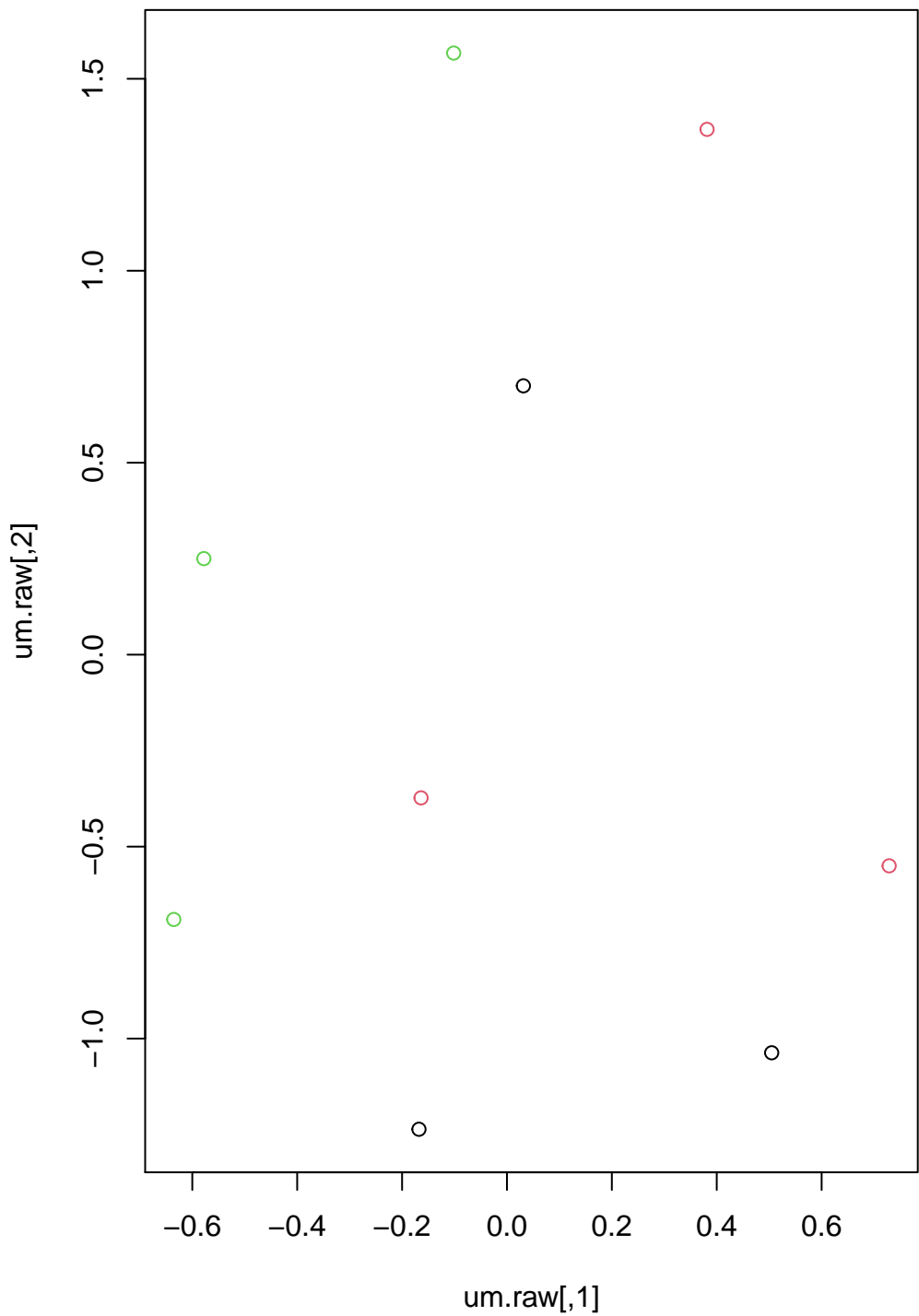

Supplement: Supplementary file 4 — Supplementary file4 (PDF 5 KB) [file 335_2023_10026_MOESM4_ESM.pdf]

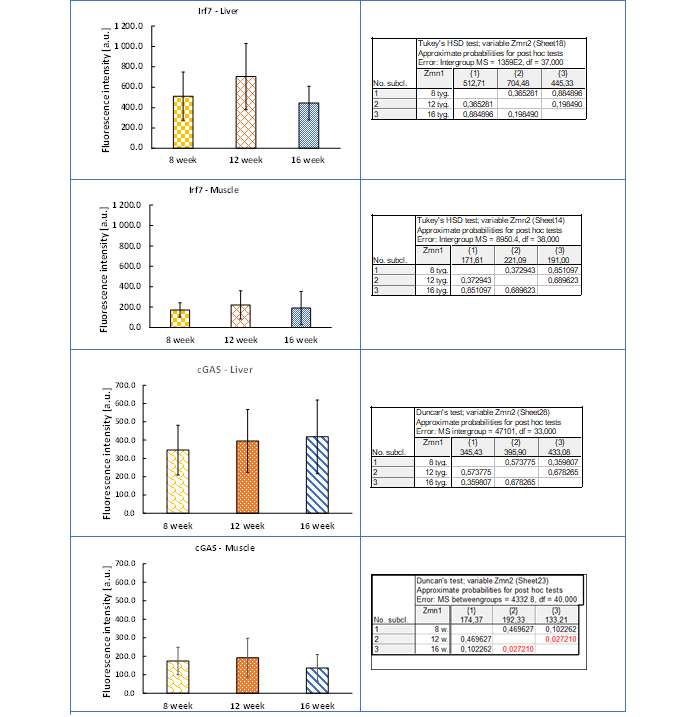

Supplement: Supplementary file 5 — Supplementary file5 (PNG 67 KB) [file 335_2023_10026_MOESM5_ESM.png]

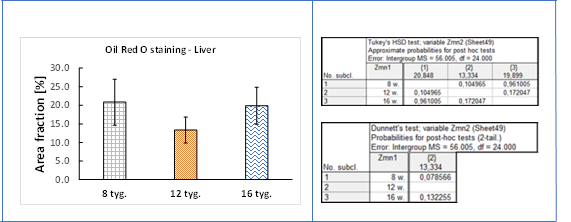

Supplement: Supplementary file 6 — Supplementary file6 (PNG 41 KB) [file 335_2023_10026_MOESM6_ESM.png]
