# Supplementary material for: Cytosolic nucleic acid sensing and mitochondrial transcriptomic changes as early triggers of metabolic disease in db/db mice
Source: Mamm Genome. 2023 Nov 18;35(1):68–76. doi: 10.1007/s00335-023-10026-z (PMC10884043; doi:10.1007/s00335-023-10026-z)
Supplement: Supplementary file 7 — Supplementary file7 (PDF 507 KB) [file 335_2023_10026_MOESM7_ESM.pdf]

Weight and glycemia (fasting conditions) measurements

				quarantine					EXPERIMENT																	
			DATE	6 weeks					8 weeks								12 weeks								16 weeks	
GROUP	SPECIES	SEX	MOUSE ID	weight [g]	weight [g]	glucose [mg/dl]	weight [g]	glucose [mg/dl]	weight [g]	glucose [mg/dl]	weight [g]	glucose [mg/dl]	weight [g]	glucose [mg/dl]	weight [g]	glucose [mg/dl]	weight [g]	glucose [mg/dl]	weight [g]	glucose [mg/dl]	weight [g]	glucose [mg/dl]	weight [g]	glucose [mg/dl]	weight [g]	glucose [mg/dl]
Group I	Dock7<m>+/+Lepr<db>/J	♂	A	34,0	39,30	311	41,59	336	43,70	315																
			B	33,0	33,80	192	35,54	373	39,30	143																
			C	32,5	32,70	173	36,42	246	39,90	216																
Group II		♂	D	31,0	36,20	202	39,62	230	42,00	230	43,90	259	45,80	284	48,10	446	50,50	345								
			E	30,0	34,30	180	37,97	213	40,20	194	41,40	143	43,20	169	44,60	175	45,30	114								
			F	28,0	33,00	281	33,95	189	36,00	201	37,80	184	41,90	164	45,10	225	47,40	219								
Group III		♂	G	28,0	29,60	122	33,27	162	36,30	140	39,50	185	41,20	161	42,80	186	45,41	178	47,81	332	48,75	404	48,79	343	49,40	262
			H	26,0	26,30	124	27,48	127	28,70	147	29,70	175	31,60	137	34,00	160	37,08	156	39,94	144	40,97	176	39,41	135	38,22	106
			I	22,0	24,20	128	25,84	150	27,30	108	29,40	128	31,30	125	33,20	140	34,70	138	36,03	132	36,60	176	36,64	164	38,43	135
