# Supplementary material for: Cytosolic nucleic acid sensing and mitochondrial transcriptomic changes as early triggers of metabolic disease in db/db mice
Source: Mamm Genome. 2023 Nov 18;35(1):68–76. doi: 10.1007/s00335-023-10026-z (PMC10884043; doi:10.1007/s00335-023-10026-z)
Supplement: Supplementary file 8 — Supplementary file8 (PDF 393 KB) [file 335_2023_10026_MOESM8_ESM.pdf]

	Group I - 8 week old mice			Group II - 12 week old mice			Group III - 16 week old mice		
**Mouse ID**	**A**	**B**	**C**	**D**	**E**	**F**	**G**	**H**	**I**
**ALT [U/l]**	171	198	160	255	42	206	171	213	183
**AspAT [U/l]**	650	530	549	302	345	428	730	866	1050
**Total cholesterol [mg/dl]**	290	210	210	130	140	180	90	70	180
**HDL cholesterol [mg/dl]**	157	148	137	99	105	116	86	64	109
**LDL cholesterol [mg/dl]**	na	2	21.4	3.8	0.6	24.2	na	na	na
**Triglycerides [mg/dl]**	601	300	258	136	172	199	208	215	430
